# In Vitro Activity of Imipenem-Funobactam Against Clinical Isolates of Gram-Negative Bacilli

**DOI:** 10.3390/antibiotics15080733

**Published:** 2026-07-29

**Authors:** James A. Karlowsky, Mark G. Wise, Qifeng Shi, Wenming Zhang, Meijie Le, Jianguo Li

**Affiliations:** 1 IHMA, Schaumburg, IL 60173, USA; jkarlowsky@sharedhealthmb.ca; 2 Department of Medical Microbiology and Infectious Diseases, Max Rady College of Medicine, University of Manitoba, Winnipeg, MB R3E 3J7, Canada; 3 Evopoint Biosciences Co., Ltd., Suzhou 215000, China; qifeng.shi@evopointbio.com (Q.S.); wenming.zhang@evopointbio.com (W.Z.); jason.le@evopointbio.com (M.L.); james.li@evopointbio.com (J.L.)

**Keywords:** funobactam, XNW4107, imipenem, Gram-negative, carbapenem-resistant

## Abstract

**Objectives**: The intent of this study was to report reference in vitro antimicrobial susceptibility testing results for funobactam (formerly XNW4107) in combination with imipenem against recent, worldwide clinical isolates of Gram-negative bacilli. **Methods**: MICs for imipenem in combination with a fixed concentration of funobactam (8 mg/L), and seven comparator agents, were determined using the reference CLSI M07 broth microdilution method for 4003 clinical isolates of Gram-negative bacilli (2008 Enterobacterales, 999 *Acinetobacter baumannii*, and 996 *Pseudomonas aeruginosa*) collected from 211 unique clinical laboratory sites in 54 countries as part of industry-sponsored antimicrobial surveillance studies in 2021 and 2022. MICs were interpreted by 2026 CLSI M100 breakpoints. Most isolates with imipenem-funobactam MICs of ≥4 mg/L (95.4%) underwent whole genome sequencing to identify acquired β-lactamase gene carriage. **Results**: MIC_90_ values for imipenem-funobactam were 2 mg/L for all 2008 isolates of Enterobacterales, and 1, 0.5, and 0.5 mg/L, respectively, for metallo-β-lactamase (MBL)-negative Enterobacterales (*n* = 1969), non-*Morganellaceae* Enterobacterales (NME) (*n* = 1752), and MBL-negative NME (*n* = 1718) isolate subsets. MIC_90_ values for imipenem-funobactam and imipenem alone were identical or within one doubling dilution for all 14 species of Enterobacterales tested except *Klebsiella pneumoniae*, *Citrobacter freundii*, and *Enterobacter bugandensis* which showed differences of 32-fold (imipenem-funobactam MIC_90_, 0.5 mg/L; imipenem MIC_90_, 16 mg/L), 8-fold (0.25 mg/L; 2 mg/L), and 4-fold (0.25 mg/L; 1 mg/L), respectively. Both for all *A. baumannii* isolates (*n* = 999) and for MBL-negative isolates (*n* = 962), the imipenem-funobactam MIC_90_ value (4 mg/L) was 32-fold lower than for imipenem alone (128 mg/L). Both for all *P. aeruginosa* (*n* = 996) isolates and MBL-negative isolates (*n* = 962), we observed a 4-fold difference in potency between imipenem-funobactam (MIC_90_, 4 mg/L) and imipenem alone (MIC_90_, 16 mg/L). Isolates carrying MBL demonstrated the highest MICs for imipenem-funobactam. **Conclusions**: Imipenem-funobactam demonstrated potent in vitro activity against most carbapenem-resistant isolates of *A. baumannii*, *K. pneumoniae*, NME and many isolates of carbapenem-resistant *P. aeruginosa*. Continued development of imipenem-funobactam is warranted.

## 1. Introduction

Antimicrobial resistance is a global public health emergency. The prevalence of Gram-negative bacilli with carbapenem-resistant and difficult-to-treat resistance (DTR) phenotypes are increasing globally [1,2]. The current (2024) World Health Organization (WHO) bacterial priority pathogens list includes carbapenem-resistant Enterobacterales and carbapenem-resistant *Acinetobacter baumannii* in the critical priority group and carbapenem-resistant *Pseudomonas aeruginosa* in the high priority group [3]. One approach to new agent development targeting carbapenem-resistant Gram-negative bacilli involves pairing an established β-lactam with a novel β-lactamase inhibitor.

Funobactam (formerly XNW4107) is an investigational non-β-lactam diazabicyclooctane (DBO) β-lactamase inhibitor with potent in vitro activity against Ambler class A β-lactamases, including extended-spectrum β-lactamases (ESBLs) and *Klebsiella pneumoniae* carbapenemases (KPCs), AmpC type β lactamases (Ambler class C enzymes), and OXA-48 and OXA-23 carbapenemases (Ambler class D enzymes) [4]. Other DBO β-lactamase inhibitors in marketed β-lactam-DBO β-lactamase inhibitor combinations include relebactam, which inhibits Ambler class A and C β-lactamases, avibactam, which inhibits Ambler class A, C, and OXA-48 β-lactamases, and durlobactam, which inhibits Ambler class A, C, and D β-lactamases. Vaborbactam is a non-β-lactam, cyclic boronic acid inhibitor that inhibits Ambler class A and C β-lactamases. Imipenem-funobactam, ceftazidime-avibactam, imipenem-relebactam, sulbactam-durlobactam, and meropenem-vaborbactam are inactive against metallo-β-lactamases (MBLs; Ambler class B enzymes). Cefiderocol, a siderophore-containing cephalosporin, generally inhibits all carbapenemases in Ambler classes A, B, and D; however, emerging resistance, particularly among NDM-1- and NDM-5-producing isolates, has been reported [5].

Previous publications have described the in vitro and in vivo antibacterial activity of imipenem-funobactam, including against carbapenem-resistant Gram-negative bacilli [4,6]. In one study, Li et al., using the CLSI broth microdilution method [7], reported that imipenem in combination with funobactam at a fixed concentration of 8 mg/L exhibited robust in vitro activity against carbapenem-resistant isolates of *K. pneumoniae*, *A. baumannii*, and *P. aeruginosa* [4]. In another study, Frantoni et al. reported that for selected isolates of highly imipenem-resistant (MIC > 32 mg/L) *A. baumannii*, *P. aeruginosa*, and *K. pneumoniae*, imipenem-funobactam reduced the imipenem MIC by up to 256-fold [6]. Imipenem-funobactam has been reported to be safe, well-tolerated, and to demonstrate excellent pharmacokinetic properties and adequate penetration into lung epithelial lining fluid in healthy human volunteers [8,9]. Currently, imipenem/cilastatin-funobactam is in clinical development as a parenteral agent intended for the treatment of patients with hospital-acquired bacterial pneumonia (HABP) and ventilator-associated bacterial pneumonia (VABP) (NCT05204563) [10]. Imipenem-funobactam has the potential to treat patients infected with three major carbapenem-resistant organisms, specifically, carbapenem-resistant *K. pneumoniae*, *A. baumannii*, and *P. aeruginosa*.

Of note, *Morganellaceae*, a family within the order Enterobacterales that includes *Proteus* species, *Providencia* species, and *Morganella morganii*, demonstrates intrinsic resistance to imipenem by a β-lactamase-independent mechanism [11]. This same spectral limitation also applies to imipenem-DBO β-lactamase inhibitor combinations such as imipenem-funobactam and imipenem-relebactam. Fortunately, species of *Morganellaceae* remain infrequent Gram-negative pathogens among hospitalized patients with infections [12,13].

The current study evaluated the in vitro activities of imipenem-funobactam and seven relevant comparator agents against clinical isolates of Gram-negative bacilli collected globally in 2021 and 2022.

## 2. Results

### 2.1. In Vitro Activity of Imipenem-Funobactam Against Global Clinical Isolates of Gram-Negative Bacilli

Against all 2008 clinical isolates of Enterobacterales, imipenem-funobactam, imipenem alone, and imipenem-relebactam had MIC_90_s of 2 mg/L with percent susceptible values for imipenem-funobactam (89.8%; MICs interpreted using 2026 CLSI M100 breakpoints for imipenem) [11] and imipenem-relebactam (89.5%) approximately 4% higher than for imipenem alone (85.4%) (Table 1). Against MBL-negative Enterobacterales, the MIC_90_ for both imipenem-funobactam (91.6% susceptible) and imipenem-relebactam (91.3% susceptible) was 1 mg/L, one doubling dilution lower than for imipenem alone (87.1% susceptible). Against the subset of 1752 isolates of non-*Morganellaceae* Enterobacterales (NME), imipenem-funobactam, imipenem-relebactam, and imipenem alone had MIC_90_s of 0.5, 0.5, and 1 mg/L, respectively, with percent susceptible values ranging from 97.0% for imipenem-funobactam to 93.1% for imipenem alone. Against the MBL-negative subset of NME, imipenem-funobactam, imipenem-relebactam, and imipenem MIC_90_s were also 0.5, 0.5, and 1 mg/L, respectively, with percent susceptible values ranging from 99.0% for imipenem-funobactam to 94.9% for imipenem alone. Percent susceptible values for meropenem-vaborbactam (98.7%), ceftazidime-avibactam (99.1%), and cefiderocol (99.7%) approximated those of imipenem-funobactam (99.0%) against MBL-negative NME, while only 72.6% of isolates were susceptible to levofloxacin.

Against individual species of Enterobacterales, the MIC_90_ values for imipenem-funobactam and imipenem were identical or within one doubling dilution for all species except *K. pneumoniae* which showed a 32-fold difference (imipenem-funobactam MIC_90_, 0.5 mg/L; imipenem MIC_90_, 16 mg/L), *Citrobacter freundii* which showed a 8-fold difference (imipenem-funobactam MIC_90_, 0.25 mg/L; imipenem MIC_90_, 2 mg/L), and *Enterobacter bugandensis* which showed a 4-fold difference (imipenem-funobactam MIC_90_, 0.25 mg/L; imipenem MIC_90_, 1 mg/L) (Table 2). Isolates of *Proteus* spp., *Providencia* spp., and *Morganella morganii* had higher imipenem-funobactam MIC_90_s (2–4 mg/L) than other species of Enterobacterales (0.12–1 mg/L) due to the presence of intrinsic β-lactamase-independent resistance to imipenem present in many isolates of these species [11].

Cefiderocol (all isolates, 96.4% susceptible; MBL-negative isolates, 97.5% susceptible) and imipenem-funobactam (all isolates, 82.5% susceptible; MBL-negative isolates, 85.7% susceptible) were the only two agents tested that were active against *A. baumannii* (Table 1). Imipenem-relebactam, ceftazidime-avibactam, and meropenem-vaborbactam are inactive against *A. baumannii* and CLSI does not publish breakpoints for these agents [11]. Percent susceptible values for imipenem, meropenem, and levofloxacin were ≤31% for *A. baumannii*. The MIC_90_ value of imipenem-funobactam (4 mg/L) was 32-fold lower than for imipenem alone (128 mg/L) both for all isolates and for MBL-negative isolates of *A. baumannii*.

The MIC_90_ value for imipenem-funobactam (4 mg/L) was 4-fold lower than for imipenem alone (16 mg/L) both for all isolates of *P. aeruginosa* and for the 962-isolate MBL-negative subset of *P. aeruginosa*. Against all isolates of *P. aeruginosa* tested and against the MBL-negative isolate subset, imipenem-funobactam (85.5% and 88.6% susceptible) was slightly less active than imipenem-relebactam (91.2% and 94.4%), ceftazidime-avibactam (94.0% and 97.6%), and cefiderocol (99.6% and 99.8%). Meropenem-vaborbactam is inactive against *P. aeruginosa* [11].

When isolates of Enterobacterales, NME, *A. baumannii*, and *P. aeruginosa* were categorized by isolate infection source (blood, intra-abdominal, respiratory tract, skin and soft tissue, and urinary tract), percent susceptible values for imipenem-funobactam were similar (within 5%) for isolates of NME (percent susceptible range, 95.7% [respiratory tract] to 99.1% [skin and soft tissue]), while isolates of *A. baumannii* (81.0% [blood] to 87.9% [skin and soft tissue]), Enterobacterales (84.8% [skin and soft tissue] to 92.1% [intra-abdominal]), and *P. aeruginosa* (82.8% [respiratory tract] to 92.2% [skin and soft tissue]) showed wider percent susceptible ranges of up to 10% (Tables S1–S4).

### 2.2. In Vitro Activity of Imipenem-Funobactam Against Global Clinical Isolates of Carbapenem-Resistant and Difficult-to-Treat Resistance (DTR) Gram-Negative Bacilli

Table 3 summarizes the in vitro activities of imipenem-funobactam and comparator agents against carbapenem-resistant and difficult-to-treat resistance (DTR) isolates of Enterobacterales, NME, *K. pneumoniae*, *A. baumannii*, and *P. aeruginosa*. Enterobacterales, including *K. pneumoniae* and NME, were classified as carbapenem-resistant based on a meropenem and/or imipenem MIC value of ≥4 mg/L [11]. *A. baumannii* and *P. aeruginosa* isolates were classified as carbapenem-resistant based on a meropenem and/or imipenem MIC value of ≥8 mg/L [11]. DTR Gram-negative bacilli isolates were defined as those intermediate or resistant to both carbapenems tested (imipenem, meropenem) and levofloxacin (the fluoroquinolone tested) using criteria established by Kadri et al. [14]; these criteria exclude ceftazidime-avibactam, imipenem-relebactam, meropenem-vaborbactam, and cefiderocol phenotypes from the definition of DTR. Percent susceptible values for imipenem-funobactam and ceftazidime-avibactam were identical (54.2%) against carbapenem-resistant NME; imipenem-relebactam (40.6% susceptible) and meropenem-vaborbactam (45.8% susceptible) were less active than imipenem-funobactam and ceftazidime-avibactam. Against DTR NME, imipenem-funobactam (55.3% susceptible) and ceftazidime-avibactam (55.3%) demonstrated greater activity than did imipenem-relebactam (40.0%) and meropenem-vaborbactam (43.5%). Percent susceptible values for imipenem-funobactam (59.7%) and ceftazidime-avibactam (58.3%) were similar against carbapenem-resistant *K. pneumoniae*; imipenem-relebactam (40.3% susceptible) and meropenem-vaborbactam (43.1% susceptible) were less active than imipenem-funobactam and ceftazidime-avibactam. Against DTR *K. pneumoniae,* imipenem-funobactam (60.3% susceptible) and ceftazidime-avibactam (58.8%) demonstrated greater activity than did imipenem-relebactam (39.7%) and meropenem-vaborbactam (42.6%).

Imipenem-funobactam inhibited 75% of both carbapenem-resistant and DTR isolates of *A. baumannii*. Imipenem-funobactam showed variable activity against carbapenem-resistant (37.9% susceptible) and DTR (16.0%) *P. aeruginosa*; imipenem-funobactam was less active than imipenem-relebactam and ceftazidime-avibactam against these resistant isolate subsets of *P. aeruginosa*.

### 2.3. In Vitro Activity of Imipenem-Funobactam Against Global Carbapenem-Resistant Gram-Negative Bacilli Stratified by Acquired β-Lactamase Carriage

**Figure 1:**
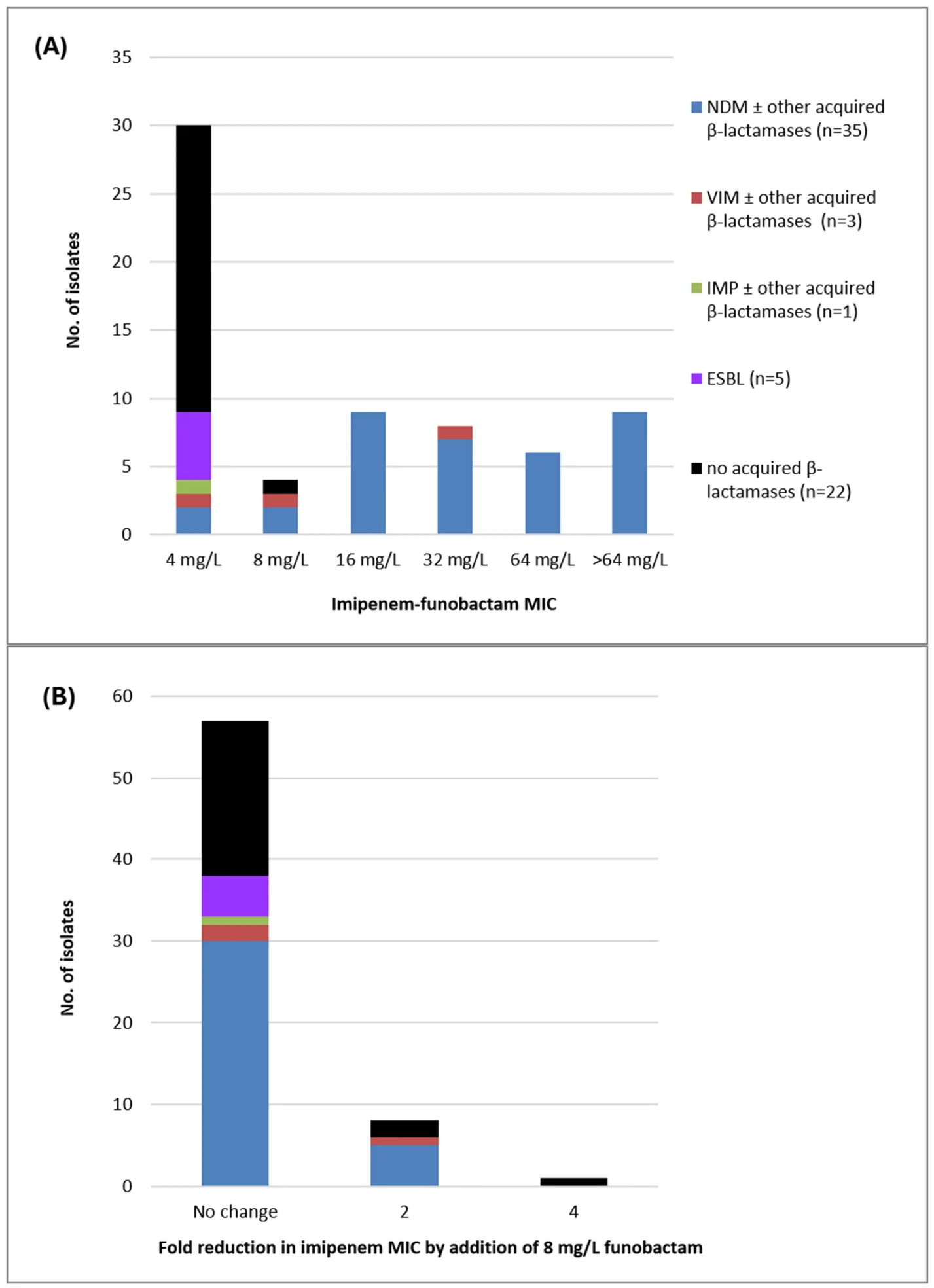
(**A**) Imipenem-funobactam MIC value distribution and (**B**) fold reduction in imipenem MIC by addition of 8 mg/L funobactam for the 66 sequenced isolates of Enterobacterales with imipenem-funobactam MICs ≥ 4 mg/L grouped by acquired β-lactamase gene carriage.

Most isolates testing with an imipenem-funobactam MIC ≥ 4 mg/L (374/392, 95.4%) were queried for β-lactamase gene carriage by whole genome sequencing. For Enterobacterales, 73 isolates had imipenem-funobactam MIC ≥ 4 mg/L, of which 66 (90.4%) were successfully sequenced (Figure 1A); 21 of 22 isolates (95.5%) with no identified acquired β-lactamase gene were *Morganellaceae* (*Proteus*, *Providencia*, *Morganella*) which demonstrate intrinsic resistance to imipenem by a mechanism other than β-lactamase production [11]. Thirty-nine of the remaining 44 isolates (88.6%) carried an MBL (35 NDM, 3 VIM, and 1 IMP). Isolates carrying MBLs had the highest imipenem-funobactam MICs (16–>64 mg/L). The five isolates carrying ESBLs, including two isolates with CARB-2, two isolates with CTX-M-14, and one isolate with CTX-M-2 were also all *Morganellaceae*. Overall, for Enterobacterales, funobactam had essentially no effect on imipenem MICs for isolates with imipenem-funobactam MICs ≥ 4 mg/L as 65 of the 66 isolates (which included both NME and *Morganellaceae* isolates) showed no change in MIC or at most one doubling-dilution change in MIC between imipenem-funobactam and imipenem alone (Figure 1B), within the acceptable error of plus/minus one doubling dilution for broth microdilution testing [7].

**Figure 2:**
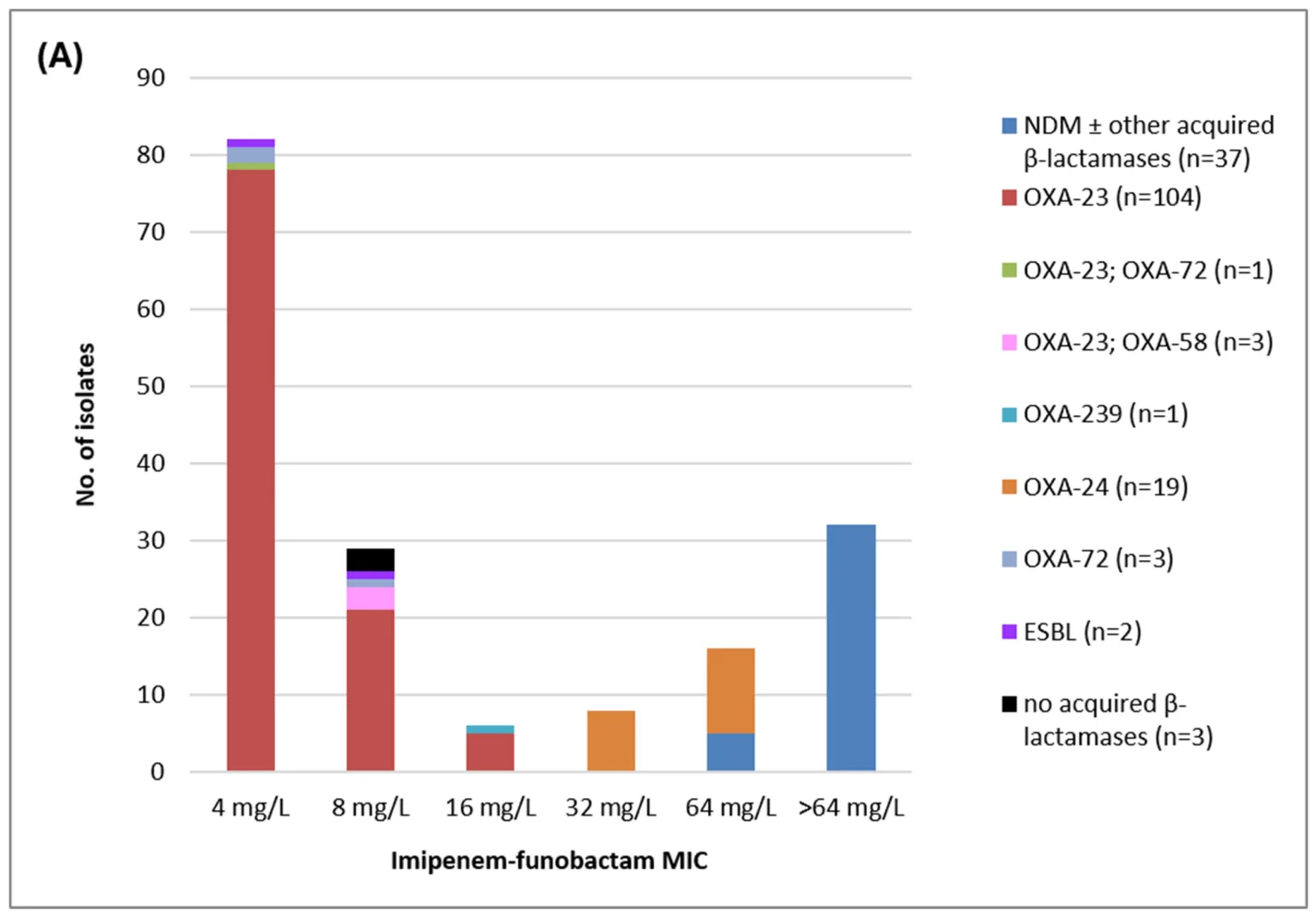

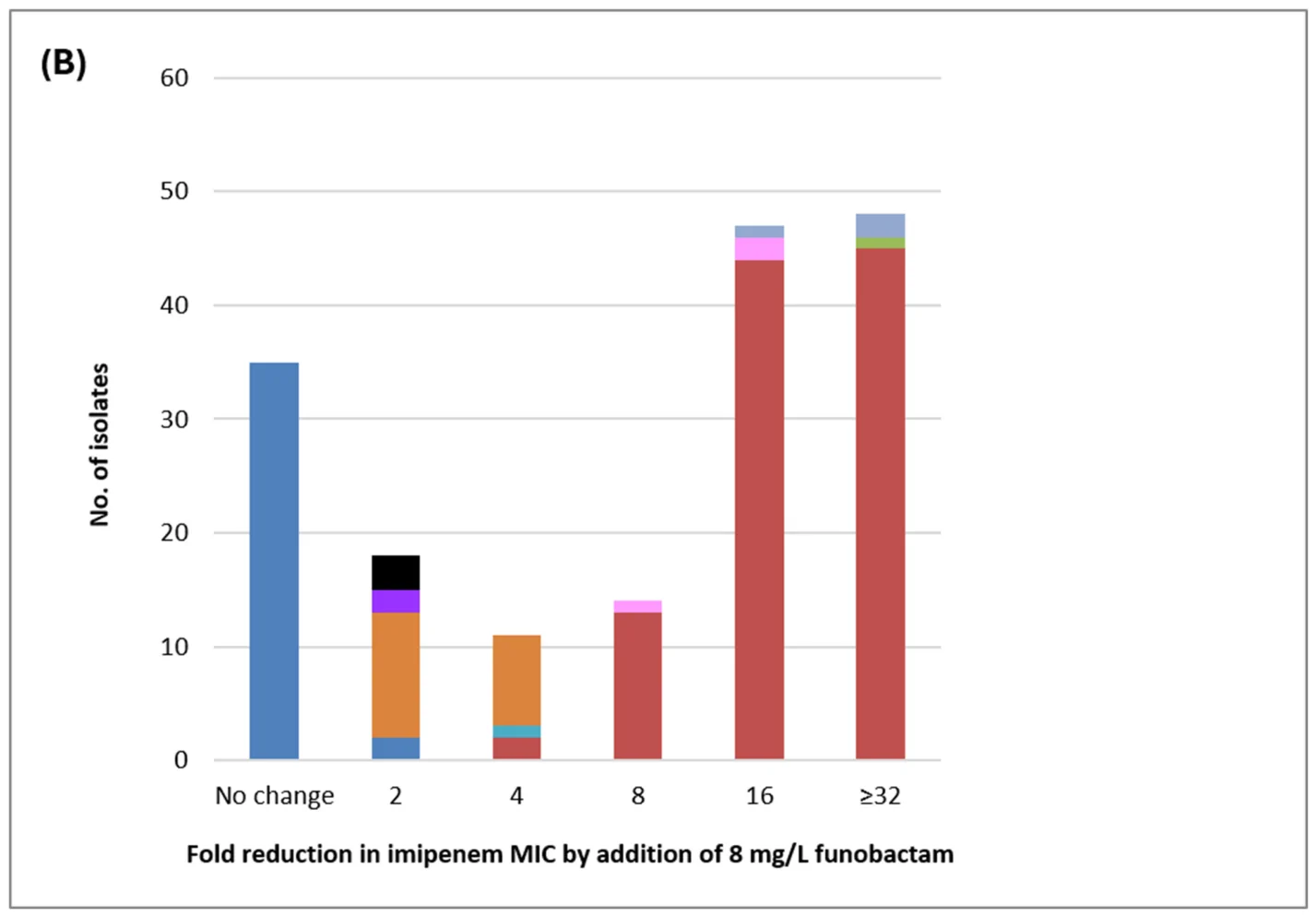
(**A**) Imipenem-funobactam MIC value distribution and (**B**) fold reduction in imipenem MIC by addition of 8 mg/L funobactam for the 173 sequenced isolates of *A. baumannii* with imipenem-funobactam MICs ≥ 4 mg/L grouped by acquired β-lactamase gene carriage.

For *A. baumannii*, 173 of the 175 (98.9%) isolates that had imipenem-funobactam MICs of ≥4 mg/L were sequenced (Figure 2A). Isolates carrying an MBL (NDM) or OXA-24 accounted for those testing with the highest MICs for imipenem-funobactam (32–>64 mg/L) with all isolates with MICs of >64 mg/L being NDM-positive. The two ESBL-positive isolates carried SHV-12. Funobactam had its greatest effect on imipenem MICs for isolates of *A. baumannii* carrying OXA-23 with imipenem-funobactam MICs 4 to ≥32-fold lower than for imipenem alone (Figure 2B) indicating that the presence of OXA-23 likely did not contribute in a meaningful way to imipenem-funobactam MICs ≥ 4 mg/L. As for Enterobacterales, *A. baumannii* isolates carrying an MBL gene were unaffected by the addition of funobactam to imipenem.

**Figure 3:**
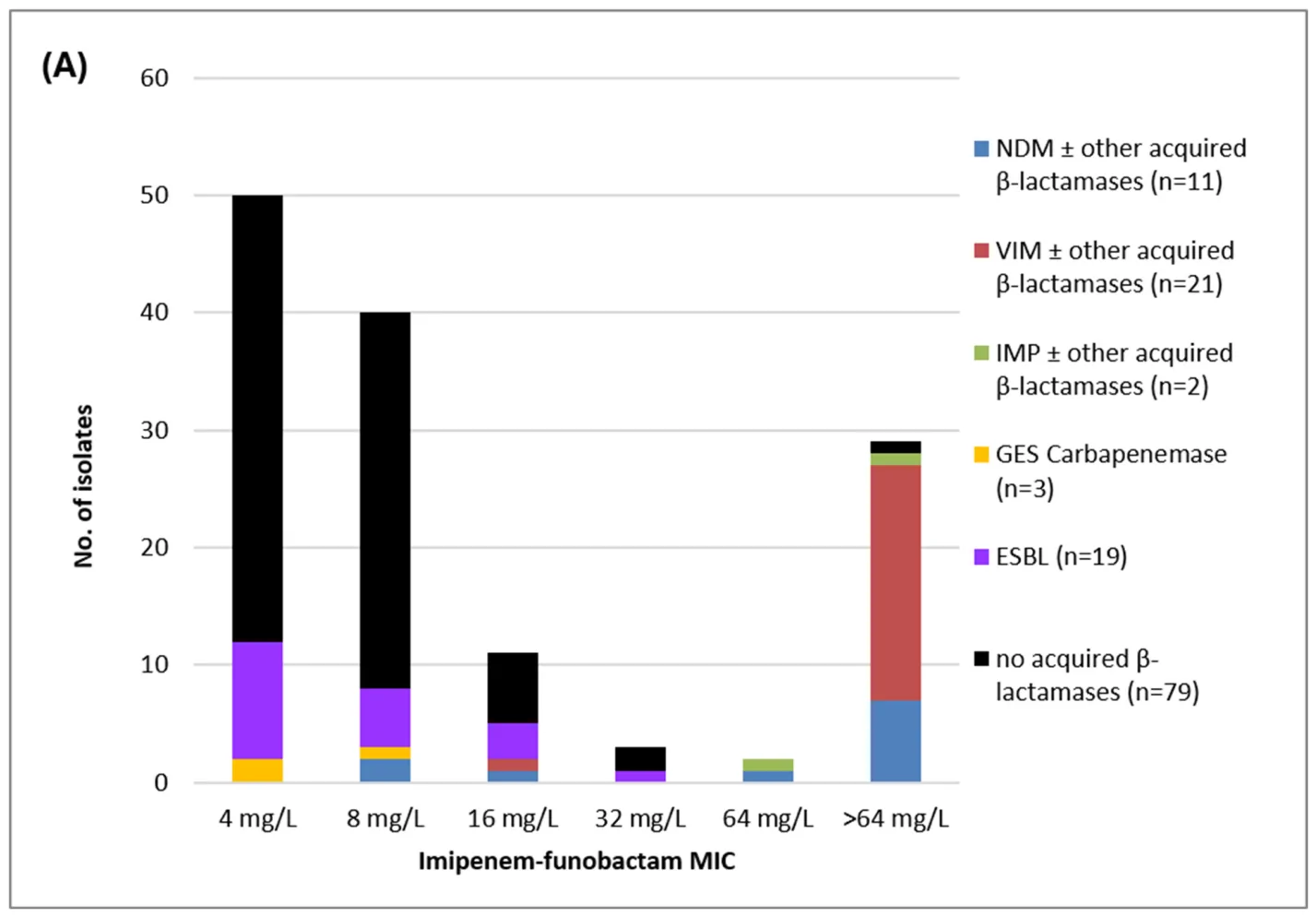

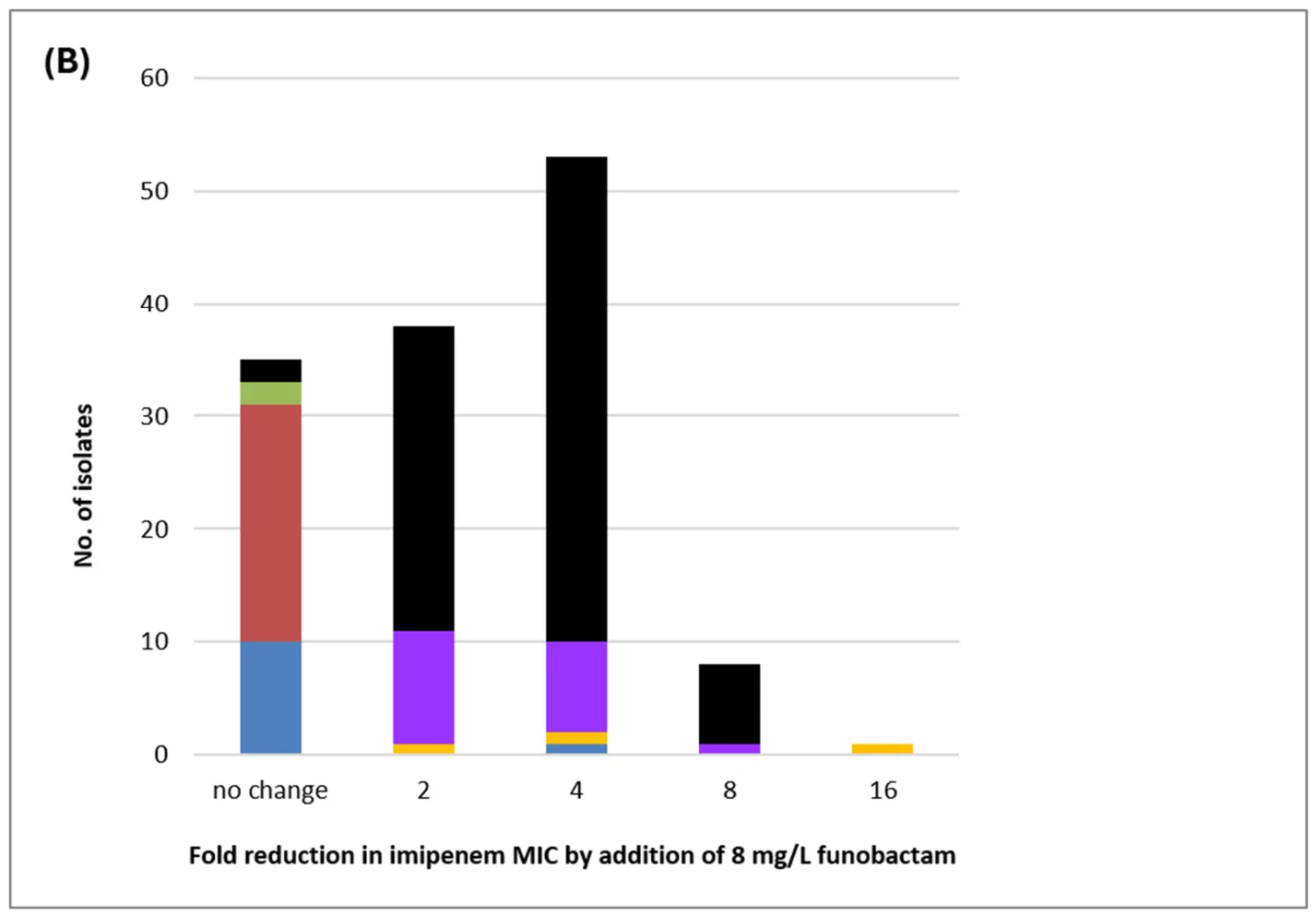
(**A**) Imipenem-funobactam MIC value distribution and (**B**) fold reduction in imipenem MIC by addition of 8 mg/L funobactam for the 135 sequenced isolates of *P. aeruginosa* with imipenem-funobactam MICs ≥ 4 mg/L grouped by acquired β-lactamase gene carriage.

For *P. aeruginosa*, 135 of the 144 (93.8%) isolates that had imipenem-funobactam MICs of ≥4 mg/L were sequenced (Figure 3A). Isolates not carrying an acquired β-lactamase gene accounted for 58.5% (79/135) of isolates with an imipenem-funobactam MIC of ≥4 mg/L. The three GES carbapenemase genes identified were GES-5, GES-6, and GES-28. The 19 ESBL-positive isolates included Class A and Class D ESBLs, specifically, CARB-2 (*n* = 2), CARB-2 and OXA-9 (*n* = 1), CTX-M-2 and OXA-129 (*n* = 2), CTX-M-2 (*n* = 1), OXA-1 (*n* = 2), OXA-2 (*n* = 5), OXA-10 (*n* = 2), OXA-1 and PER-3 (*n* = 1), OXA-35 (*n* = 1), and GES-1 (*n* = 2). *P. aeruginosa* carrying an MBL gene accounted for most isolates of those testing with the highest MICs for imipenem-funobactam (64 and >64 mg/L). Funobactam lowered the imipenem MICs of many isolates not carrying an acquired β-lactamase suggesting that it inhibited PDC, the AmpC present in all isolates of *P. aeruginosa* (Figure 3B). The addition of funobactam to imipenem had no effect on the MICs of isolates carrying an MBL gene.

## 3. Discussion

Imipenem combined with funobactam at a fixed concentration of 8 mg/L demonstrated potent in vitro activity against recent (collected in 2021 and 2022) global clinical isolates of Enterobacterales (*n* = 2008), *A. baumannii* (*n* = 999), and *P. aeruginosa* (*n* = 996) with the greatest fold reductions in imipenem MICs observed for *K. pneumoniae* (32-fold) and *A. baumannii* (32-fold) (Table 1 and Table 2). The imipenem-funobactam MIC_90_s were 2, 0.5, 4, and 4 mg/L, respectively, for all isolates of Enterobacterales, NME, *A. baumannii*, and *P. aeruginosa* tested. When MBL-producing isolates were removed from each of those four organism groups, the imipenem-funobactam MIC_90_s were 1, 0.5, 4, and 4 mg/L, respectively, for Enterobacterales, NME, *A. baumannii*, and *P. aeruginosa*.

Previously, Li et al. tested 54 isolates of imipenem-resistant *K. pneumoniae* collected across China from 2017 to 2019 against imipenem combined with funobactam at a fixed concentration of 8 mg/L, and reported an MIC_90_ of 2 mg/L, a 128-fold reduction in MIC_90_ compared to imipenem alone with 88.9% of isolates at or below the CLSI imipenem-susceptible breakpoint for Enterobacterales of ≤1 mg/L [4]. In the current study of 520 *K. pneumoniae* isolates, we observed an imipenem-funobactam MIC_90_ of 0.5 mg/L (94.4% susceptible) in the context of 13.8% imipenem-resistant isolates (Table 2). Against the 72 imipenem (carbapenem)-resistant *K. pneumoniae* isolates, the percent susceptible values for imipenem-funobactam (MIC_90_, 64 mg/L), ceftazidime-avibactam (MIC_90_, >128 mg/L), meropenem-vaborbactam (MIC_90_, >64 mg/L), and imipenem-relebactam (MIC_90_, >64 mg/L), were 59.7%, 58.3%, 43.1%, and 40.3%, respectively (Table 3). Of note, 27 of the 72 imipenem (carbapenem)-resistant *K. pneumoniae* isolates exhibited imipenem-funobactam MIC values ≥ 4 mg/L and were sequenced. Among these, all but one (26/27, 96.3%) carried MBLs, against which these β-lactam/β lactamase combinations are inactive. After removing the MBL-producing isolates, the percent susceptible values of imipenem (carbapenem)-resistant *K. pneumoniae* against imipenem-funobactam (MIC_90_, 1 mg/L), ceftazidime-avibactam (MIC_90_, 8 mg/L), meropenem-vaborbactam (MIC_90_, 32 mg/L), and imipenem-relebactam (MIC_90_, 8 mg/L) were 93.5%, 91.3%, 65.2%, and 63.0%, respectively.

Li et al. also tested imipenem-funobactam against 106 isolates of imipenem-nonsusceptible *A. baumannii* and reported an MIC_90_ of 8 mg/L, which was 16-fold lower than the MIC_90_ of imipenem [4]. The current study of 999 *A. baumannii* isolates resulted an imipenem-funobactam MIC_90_ of 4 mg/L, with 69.6% of isolates tested being imipenem resistant (Table 1). Furthermore, Li et al. tested 101 isolates of imipenem-nonsusceptible *P. aeruginosa* and reported that 80% of isolates exhibited MICs of imipenem-funobactam from 2 to 8 mg/L, which were 4- to 8-fold lower than their respective MICs for imipenem [4]. In the current study of 996 *P. aeruginosa* isolates, we found an imipenem-funobactam MIC_90_ of 4 mg/L and that 85.5% of isolates were susceptible to imipenem-funobactam, slightly lower than that for imipenem-relebactam (91.2%) (Table 1). Of importance, imipenem-funobactam (MIC_90_, 8 mg/L) inhibited 75% of both carbapenem-resistant and DTR isolates of *A. baumannii*, while ceftazidime-avibactam (MIC_90_, >128 mg/L), meropenem-vaborbactam (MIC_90_, >64 mg/L), and imipenem-relebactam (MIC_90_, >64 mg/L) were all inactive against carbapenem-resistant and DTR isolates of *A. baumannii*.

Increased isolation of carbapenem-resistant Enterobacterales, *A. baumannii*, and *P. aeruginosa* is of global concern [3]. Carbapenemases are frequent causes of carbapenem resistance in Gram-negative bacilli; however, other non-β-lactamases-mediated mechanisms can also, although generally less frequently, contribute to carbapenem resistance and therefore, also deserve mention. These mechanisms often appear in combination, and include loss or reduced expression of porins, changes in expression or mutations in the genes encoding penicillin-binding proteins (e.g., PBP3 mutations), AmpC and ESBLs, and efflux pumps [15,16]. For example, ceftazidime-avibactam resistance in MBL-negative Gram-negative bacilli has been shown to result from amino acid substitutions in the Ω-loop of AmpC which reduces the binding affinity of avibactam and its inhibition of AmpC [15]. Investigation of carbapenem-resistant isolates for the presence of non-β-lactamase-based mechanisms of resistance was beyond the scope of the current study.

DTR in Gram-negative bacilli is an increasingly important consideration in empirical treatment decisions and, although less common for Enterobacterales (current study, 4.5% [90/2008]), can account for >10% of *P. aeruginosa* clinical isolates (current study, 12.6% [125/996]) and >50% of *A. baumannii* clinical isolates (current study, 68.5% [684/999]) in some patient populations [14,17,18,19]. The current study demonstrated that imipenem-funobactam was particularly active against DTR isolates of *A. baumannii* (75% susceptible) (Table 3).

Limitations of this study include that it used selected bacterial species from patients with specific infection types, and therefore, it may not reflect real resistance rates to the agents tested. Molecular testing of bacterial isolates used in this study was limited and focused solely on the presence of β-lactamase genes. Given that only isolates with imipenem-funobactam MICs ≥ 4 mg/L were molecularly characterized, not all imipenem-intermediate and imipenem-resistant isolates were sequenced. Sequencing a subset of imipenem-susceptible isolates may have provided a baseline for assessing the results for isolates with imipenem-funobactam MICs ≥ 4 mg/L.

We conclude that imipenem-funobactam provides broader in vitro coverage of key pathogenic carbapenem-resistant Gram-negative bacilli (Enterobacterales, *A. baumannii*, and *P. aeruginosa*) than newer, marketed β-lactam-β-lactamase inhibitor combinations (i.e., ceftazidime-avibactam, imipenem-relebactam, meropenem-vaborbactam, aztreonam-avibactam, and sulbactam-durlobactam) which do not target all three carbapenem-resistant Gram-negative pathogens [20,21]. Aztreonam-avibactam, ceftazidime-avibactam, imipenem-relebactam, and meropenem-vaborbactam are all inactive against *A. baumannii*; meropenem-vaborbactam is also inactive against *P. aeruginosa*. Funobactam is a strong inhibitor of OXA-23, a common Ambler class D carbapenemase carried by *A. baumannii*; in the current study, funobactam lowered imipenem MICs for isolates of *A. baumannii* carrying OXA-23 by 4 to ≥32-fold compared to imipenem alone (Figure 2B). Continued development of imipenem-funobactam is warranted given that it demonstrated potent in vitro activity against current, global, carbapenem-resistant, and DTR clinical isolates of Enterobacterales, *A. baumannii*, and *P. aeruginosa*.

## 4. Materials and Methods

### 4.1. Bacterial Isolates

In total, 4003 isolates of Gram-negative bacilli were tested; isolates were limited to one isolate per species per patient. The isolate collection included 2008 Enterobacterales, 999 *A. baumannii*, and 996 *P. aeruginosa* from the years 2021 and 2022. Isolates were randomly selected for testing from the frozen global surveillance study stock collection maintained by IHMA (Schaumburg, IL, USA). Each isolate was identified by MALDI-TOF mass spectrometry (Bruker, Billerica, MA, USA). The 2008 isolates of Enterobacterales comprised 645 *E. coli* (32.1% of Enterobacterales), 520 *K. pneumoniae* (25.9%), 171 *E. cloacae* (8.5%), 118 *K. oxytoca* (5.9%), 83 *S. marcescens* (4.1%), 81 *K. aerogenes* (4.0%), 78 *P. mirabilis* (3.9%), 59 *M. morganii* (2.9%), 51 *C. freundii* (2.5%), 43 *E. bugandensis* (2.1%), 40 *C. koseri* (2.0%), 40 *P. vulgaris* (2.0%), 40 *P. stuartii* (2.0%), and 39 *P. rettgeri* (1.9%). Geographical regions of isolate origin were Europe (1584 isolates/39.6% of all isolates), Asia/Pacific (713/17.8%), Latin America (684/17.1%), Middle East/Africa (522/13.0%), and North America (500/12.5%). Isolate infection sources were respiratory tract (1640 isolates/41.0% of all isolates), blood (942/23.5%), urinary tract (730/18.2%), intra-abdominal sources (395/9.9%), skin and soft tissue (294/7.3%), and other sources (2/0.05%).

### 4.2. Antimicrobial Susceptibility Testing

MICs were determined at IHMA using the reference CLSI broth microdilution method [7,11]. MICs for imipenem-funobactam were determined using doubling dilutions of imipenem with a fixed concentration of funobactam of 8 mg/L. Avibactam was tested at a fixed concentration of 4 mg/L in combination with doubling dilutions of ceftazidime, relebactam was tested at a fixed concentration of 4 mg/L in combination with doubling dilutions of imipenem, and vaborbactam was tested at a fixed concentration of 8 mg/L in combination with meropenem as specified in the 2026 CLSI M100 performance standard document [11]. MICs were interpreted using 2026 CLSI M100 breakpoints [11]. Imipenem-funobactam MICs were interpreted using 2026 CLSI M100 imipenem susceptible, intermediate, and resistant breakpoints as provisional breakpoints. Enterobacterales were classified as carbapenem-resistant based on a meropenem and/or imipenem MIC value of ≥4 mg/L. *A. baumannii* and *P. aeruginosa* isolates were classified as carbapenem-resistant based on a meropenem and/or imipenem MIC value of ≥8 mg/L.

### 4.3. Molecular Characterization of Isolates with Imipenem-Funobactam MICs of ≥4 mg/L

Isolates testing with imipenem-funobactam MICs of ≥4 mg/L were characterized for β-lactamase gene carriage by whole genome sequencing on an Illumina (San Diego, CA, USA) NovaSeq platform with a 2 × 150 bp read configuration. Analysis was carried out using the CLC Genomics Workbench, version 23.0.4 from Qiagen (Aarhus, Denmark). Reads were sampled to a calculated coverage depth of 100×, trimmed for quality and adapter sequences, and assembled de novo. The quality of assemblies was assessed using the CheckM lineage workflow [22,23,24]; thresholds of ≤5% contamination and ≥95% completeness were used as acceptability criteria. In total, 66 of 73 (90.4%) isolates of Enterobacterales, 173 of 175 (98.9%) isolates of *A. baumannii*, and 135 of 144 (93.8%) isolates of *P. aeruginosa* with imipenem-funobactam MICs of ≥4 mg/L met these quality criteria. Acceptable assemblies were queried for resistance genes against the Resfinder database [25] using thresholds of ≥72% and ≥35% for identity and coverage, respectively. Variants for positively identified β-lactamase genes were obtained by pairwise alignment to amino acid sequences present in the NCBI Bacterial Antimicrobial Resistance Reference Gene Database (Bioproject 313047). For *A. baumannii*, TEM-1 β-lactamase was not included in genotype groupings because ampicillin-sulbactam was not tested; TEM-1 hydrolyses sulbactam but not third-generation cephalosporins, carbapenems, or cefiderocol.

## Figures and Tables

**Table 1:** In vitro activities of imipenem-funobactam and comparator agents against global clinical isolates of Gram-negative bacilli collected in 2021 and 2022.

		MIC, mg/L			MIC Interpretation		
Organism, Genotype (n)	Antimicrobial Agent	MIC_50_	MIC_90_	MIC Range	% S	% I	% R
Enterobacterales, all isolates (2008)	Imipenem-funobactam ^a^	0.12	2	≤0.03–>64	89.8	6.5	3.6
	Imipenem	0.25	2	≤0.06–>128	85.4	6.6	8.0
	Imipenem-relebactam	0.12	2	≤0.03–>64	89.5	6.1	4.4
	Meropenem	≤0.06	0.12	≤0.06–>64	94.8	0.4	4.8
	Meropenem-vaborbactam	0.03	0.06	≤0.008–>64	97.2	0.4	2.4
	Ceftazidime-avibactam	0.12	0.5	≤0.06–>128	97.3	NA	2.7
	Cefiderocol	0.12	1	≤0.06–>32	99.7	0.2	0.1
	Levofloxacin	0.06	16	≤0.015–>16	71.4	5.9	22.7
Enterobacterales, MBL-negative (1969)	Imipenem-funobactam	0.12	1	≤0.03–64	91.6	6.7	1.7
	Imipenem	0.25	2	≤0.06–>128	87.1	6.8	6.1
	Imipenem-relebactam	0.12	1	≤0.03–>64	91.3	6.1	2.5
	Meropenem	≤0.06	0.12	≤0.06–>64	96.6	0.4	2.9
	Meropenem-vaborbactam	0.03	0.06	≤0.008–>64	98.9	0.3	0.8
	Ceftazidime-avibactam	0.12	0.5	≤0.06–>128	99.2	NA	0.8
	Cefiderocol	0.12	0.5	≤0.06–>32	99.7	0.2	0.1
	Levofloxacin	0.06	16	≤0.015–>16	72.6	5.8	21.6
NME ^b^, all isolates (1752)	Imipenem-funobactam	0.12	0.5	≤0.03–>64	97.0	0.6	2.4
	Imipenem	0.25	1	≤0.06–>128	93.1	1.4	5.5
	Imipenem-relebactam	0.12	0.5	≤0.03–>64	96.3	0.7	3.0
	Meropenem	≤0.06	0.12	≤0.06–>64	94.3	0.5	5.2
	Meropenem-vaborbactam	0.03	0.06	≤0.008–>64	97.0	0.4	2.6
	Ceftazidime-avibactam	0.12	1	≤0.06–>128	97.2	NA	2.8
	Cefiderocol	0.12	1	≤0.06–>32	99.7	0.2	0.1
	Levofloxacin	0.06	16	≤0.015–>16	71.4	5.8	22.8
NME, MBL-negative (1718)	Imipenem-funobactam	0.12	0.5	≤0.03–64	99.0	0.6	0.5
	Imipenem	0.25	1	≤0.06–>128	94.9	1.5	3.6
	Imipenem-relebactam	0.12	0.5	≤0.03–>64	98.3	0.6	1.1
	Meropenem	≤0.06	≤0.06	≤0.06–>64	96.2	0.5	3.4
	Meropenem-vaborbactam	0.03	0.06	≤0.008–>64	98.7	0.3	0.9
	Ceftazidime-avibactam	0.12	0.5	≤0.06–>128	99.1	NA	0.9
	Cefiderocol	0.12	1	≤0.06–>32	99.7	0.2	0.1
	Levofloxacin	0.06	16	≤0.015–>16	72.6	5.7	21.7
*A. baumannii*, all isolates (999)	Imipenem-funobactam	1	4	0.06–>64	82.5	8.4	9.1
	Imipenem	64	128	≤0.06–>128	30.1	0.3	69.6
	Imipenem-relebactam	64	>64	0.06–>64	NA	NA	NA
	Meropenem	64	>64	≤0.06–>64	29.7	0.3	70.0
	Meropenem-vaborbactam	64	>64	≤0.008–>64	NA	NA	NA
	Ceftazidime-avibactam	32	>128	0.25–>128	NA	NA	NA
	Cefiderocol	0.25	2	≤0.06–>32	96.4	0.5	3.1
	Levofloxacin	8	>16	0.03–>16	28.4	6.5	65.1
*A. baumannii*, MBL-negative (962)	Imipenem-funobactam	1	4	0.06–64	85.7	8.7	5.6
	Imipenem	64	128	≤0.06–>128	31.3	0.3	68.4
	Imipenem-relebactam	64	>64	0.06–>64	NA	NA	NA
	Meropenem	64	>64	≤0.06–>64	30.9	0.3	68.8
	Meropenem-vaborbactam	64	>64	≤0.008–>64	NA	NA	NA
	Ceftazidime-avibactam	32	128	0.25–>128	NA	NA	NA
	Cefiderocol	0.25	1	≤0.06–>32	97.5	0.2	2.3
	Levofloxacin	8	>16	0.03–>16	29.3	5.7	65.0
*P. aeruginosa*, all isolates (996)	Imipenem-funobactam	0.5	4	0.12–>64	85.5	5.5	8.9
	Imipenem	2	16	0.12–>128	73.4	4.7	21.9
	Imipenem-relebactam	0.5	2	0.12–>64	91.2	4.1	4.7
	Meropenem	0.5	8	≤0.06–>64	79.3	5.6	15.1
	Meropenem-vaborbactam	0.5	8	≤0.008–>64	NA	NA	NA
	Ceftazidime-avibactam	2	8	≤0.06–>128	94.0	NA	6.0
	Cefiderocol	0.25	0.5	≤0.06–>32	99.6	0.3	0.1
	Levofloxacin	0.5	16	≤0.015–>16	78.7	6.1	15.2
*P. aeruginosa*, MBL-negative (962)	Imipenem-funobactam	0.5	4	0.12–>64	88.6	5.7	5.7
	Imipenem	2	16	0.12–>128	76.0	4.9	19.1
	Imipenem-relebactam	0.5	2	0.12–>64	94.4	4.3	1.4
	Meropenem	0.5	8	≤0.06–>64	82.1	5.8	12.1
	Meropenem-vaborbactam	0.25	8	≤0.008–>64	NA	NA	NA
	Ceftazidime-avibactam	2	8	≤0.06–>128	97.3	NA	2.7
	Cefiderocol	0.25	0.5	≤0.06–>32	99.8	0.1	0.1
	Levofloxacin	0.25	4	≤0.015–>16	81.3	6.3	12.4

Abbreviations: % S, percent susceptible; % I, percent intermediate; % R, percent resistant; MBL, metallo-β-lactamase; NA, not applicable; NME, Non-*Morganellaceae* Enterobacterales. ^a^ Imipenem-funobactam MICs were interpreted using 2026 CLSI M100 imipenem susceptible, intermediate, and resistant breakpoints as provisional breakpoints [11]. ^b^ NME species exclude *Proteus* species, *Providencia* species, and *Morganella morganii* which demonstrate intrinsic resistance to imipenem by a β-lactamase-independent mechanism [11].

**Table 2:** In vitro activities of imipenem-funobactam and imipenem against global clinical isolates of individual species of Enterobacterales collected in 2021 and 2022.

		MIC, mg/L			MIC Interpretation		
Enterobacterales Species (n)	Antimicrobial Agent	MIC_50_	MIC_90_	MIC Range	% S	% I	% R
*Escherichia coli* (645)	Imipenem-funobactam ^a^	0.12	0.25	≤0.03–32	98.8	0.8	0.5
	Imipenem	0.12	0.25	≤0.06–32	98.4	0.8	0.8
*Klebsiella pneumoniae* (520)	Imipenem-funobactam	0.12	0.5	≤0.03–>64	94.4	0.2	5.4
	Imipenem	0.25	16	≤0.06–>128	85.0	1.2	13.8
*Enterobacter cloacae* (171)	Imipenem-funobactam	0.12	0.25	0.06–32	97.1	0.6	2.3
	Imipenem	0.25	0.5	≤0.06–32	95.3	1.2	3.5
*Klebsiella oxytoca* (118)	Imipenem-funobactam	0.12	0.25	0.12–4	99.2	0	0.8
	Imipenem	0.12	0.25	0.12–4	99.2	0	0.8
*Serratia marcescens* (83)	Imipenem-funobactam	0.5	1	0.12–32	97.6	1.2	1.2
	Imipenem	0.5	1	0.12–128	92.8	2.4	4.8
*Klebsiella aerogenes* (81)	Imipenem-funobactam	0.25	0.5	0.06–2	98.8	1.2	0
	Imipenem	0.5	1	0.12–16	92.6	3.7	3.7
*Proteus mirabilis* (78)	Imipenem-funobactam	2	4	0.12–>64	38.5	46.2	15.4
	Imipenem	2	4	0.12–128	30.8	42.3	26.9
*Morganella morganii* (59)	Imipenem-funobactam	2	2	0.12–4	20.3	78.0	1.7
	Imipenem	2	4	0.5–4	11.9	59.3	28.8
*Citrobacter freundii* (51)	Imipenem-funobactam	0.12	0.25	0.06–64	90.2	0	9.8
	Imipenem	0.5	2	0.12–64	84.3	5.9	9.8
*Enterobacter bugandensis* (43)	Imipenem-funobactam	0.12	0.25	0.06–2	97.7	2.3	0
	Imipenem	0.5	1	0.12–2	90.7	9.3	0
*Citrobacter koseri* (40)	Imipenem-funobactam	0.12	0.12	0.06–0.5	100	0	0
	Imipenem	0.12	0.12	≤0.06–0.5	100	0	0
*Proteus vulgaris* (40)	Imipenem-funobactam	2	4	0.12–4	40.0	37.5	22.5
	Imipenem	2	4	0.12–4	30.0	32.5	37.5
*Providencia stuartii* (40)	Imipenem-funobactam	1	4	0.12–32	50.0	32.5	17.5
	Imipenem	2	4	0.12–32	42.5	35.0	22.5
*Providencia rettgeri* (39)	Imipenem-funobactam	1	2	0.5–>64	66.7	28.2	5.1
	Imipenem	1	2	0.5–>128	61.5	33.3	5.1

Abbreviations: % S, percent susceptible; % I, percent intermediate; % R, percent resistant; NA, not applicable. ^a^ Imipenem-funobactam MICs were interpreted using 2026 CLSI M100 imipenem susceptible, intermediate, and resistant breakpoints as provisional breakpoints [11].

**Table 3:** In vitro activities of imipenem-funobactam and comparator agents against global clinical isolates of carbapenem-resistant and DTR Gram-negative bacilli collected in 2021 and 2022.

		MIC, mg/L			MIC Interpretation		
Organism/Resistance Phenotype (n)	Antimicrobial Agent	MIC_50_	MIC_90_	MIC Range	% S	% I	% R
Enterobacterales							
Carbapenem-resistant (160)	Imipenem-funobactam ^a^	2	32	0.06–>64	33.8	20.6	45.6
	Imipenem	8	128	4–>128	0	0	100
	Imipenem-relebactam	4	64	0.06–>64	25.6	20.0	54.4
	Meropenem	8	>64	≤0.06–>64	38.1	1.9	60.0
	Meropenem-vaborbactam	0.25	64	0.015–>64	64.4	5.6	30.0
	Ceftazidime-avibactam	1	>128	≤0.06–>128	69.4	NA	30.6
	Cefiderocol	0.25	2	≤0.06–16	98.1	1.3	0.6
	Levofloxacin	8	>16	0.03–>16	33.1	5.6	61.3
DTR (90)	Imipenem-funobactam	1	64	0.06–>64	52.2	2.2	45.6
	Imipenem	32	128	2–>128	0	2.2	97.8
	Imipenem-relebactam	4	64	0.06–>64	37.8	4.4	57.8
	Meropenem	64	>64	2–>64	0	4.4	95.6
	Meropenem-vaborbactam	8	>64	0.015–>64	41.1	8.9	50.0
	Ceftazidime-avibactam	4	>128	0.25–>128	52.2	NA	47.8
	Cefiderocol	1	2	≤0.06–16	97.8	1.1	1.1
	Levofloxacin	>16	>16	1–>16	0	7.8	92.2
NME ^b^							
Carbapenem-resistant (96)	Imipenem-funobactam	0.5	64	0.06–>64	54.2	2.1	43.8
	Imipenem	32	128	4–>128	0	0	100
	Imipenem-relebactam	4	64	0.06–>64	40.6	5.2	54.2
	Meropenem	32	>64	0.5–>64	2.1	3.1	94.8
	Meropenem-vaborbactam	8	>64	0.015–>64	45.8	7.3	46.9
	Ceftazidime-avibactam	4	>128	0.25–>128	54.2	NA	45.8
	Cefiderocol	1	4	≤0.06–16	96.9	2.1	1.0
	Levofloxacin	>16	>16	0.03–>16	12.5	6.3	81.3
DTR (85)	Imipenem-funobactam	0.5	64	0.06–>64	55.3	2.4	42.4
	Imipenem	32	128	2–>128	0	2.4	97.6
	Imipenem-relebactam	4	64	0.06–>64	40.0	4.7	55.3
	Meropenem	64	>64	2–>64	0	4.7	95.3
	Meropenem-vaborbactam	8	>64	0.015–>64	43.5	7.1	49.4
	Ceftazidime-avibactam	4	>128	0.25–>128	55.3	NA	44.7
	Cefiderocol	1	2	≤0.06–16	97.6	1.2	1.2
	Levofloxacin	>16	>16	1–>16	0	8.2	91.8
*K. pneumoniae*							
Carbapenem-resistant (72)	Imipenem-funobactam	0.5	64	0.06–>64	59.7	1.4	38.9
	Imipenem	32	128	4–>128	0	0	100
	Imipenem-relebactam	4	>64	0.06–>64	40.3	5.6	54.2
	Meropenem	64	>64	0.5–>64	1.4	2.8	95.8
	Meropenem-vaborbactam	16	>64	0.015–>64	43.1	5.6	51.4
	Ceftazidime-avibactam	4	>128	0.25–>128	58.3	NA	41.7
	Cefiderocol	1	2	≤0.06–8	98.6	1.4	0
	Levofloxacin	>16	>16	0.06–>16	5.6	8.3	86.1
DTR (68)	Imipenem-funobactam	0.5	>64	0.06–>64	60.3	1.5	38.2
	Imipenem	32	128	2–>128	0	1.5	98.5
	Imipenem-relebactam	4	>64	0.06–>64	39.7	5.9	54.4
	Meropenem	64	>64	2–>64	0	4.4	95.6
	Meropenem-vaborbactam	16	>64	0.015–>64	42.6	5.9	51.5
	Ceftazidime-avibactam	4	>128	0.25–>128	58.8	NA	41.2
	Cefiderocol	1	2	≤0.06–4	100	0	0
	Levofloxacin	>16	>16	1–>16	0	8.8	91.2
*A. baumannii*							
Carbapenem-resistant (700)	Imipenem-funobactam	2	8	0.06–>64	75.0	12.0	13.0
	Imipenem	64	128	0.25–>128	0.3	0.4	99.3
	Imipenem-relebactam	64	>64	0.25–>64	NA	NA	NA
	Meropenem	64	>64	4–>64	0	0.1	99.9
	Meropenem-vaborbactam	64	>64	≤0.008–>64	NA	NA	NA
	Ceftazidime-avibactam	64	>128	1–>128	NA	NA	NA
	Cefiderocol	0.5	2	≤0.06–>32	95.0	0.7	4.3
	Levofloxacin	16	>16	0.12–>16	2.1	8.7	89.1
DTR (684)	Imipenem-funobactam	2	8	0.06–>64	74.7	12.3	13.0
	Imipenem	64	128	4–>128	0	0.4	99.6
	Imipenem-relebactam	64	>64	2–>64	NA	NA	NA
	Meropenem	64	>64	4–>64	0	0.1	99.9
	Meropenem-vaborbactam	64	>64	≤0.008–>64	NA	NA	NA
	Ceftazidime-avibactam	64	>128	1–>128	NA	NA	NA
	Cefiderocol	0.5	2	≤0.06–>32	94.9	0.7	4.4
	Levofloxacin	16	>16	4–>16	0	8.9	91.1
*P. aeruginosa*							
Carbapenem-resistant (232)	Imipenem-funobactam	4	>64	0.25–>64	37.9	23.7	38.4
	Imipenem	16	>128	1–>128	2.2	3.9	94.0
	Imipenem-relebactam	2	>64	0.25–>64	62.1	17.7	20.3
	Meropenem	8	>64	0.5–>64	15.9	19.4	64.7
	Meropenem-vaborbactam	8	64	0.06–>64	NA	NA	NA
	Ceftazidime-avibactam	4	64	0.5–>128	76.3	NA	23.7
	Cefiderocol	0.25	1	≤0.06–>32	98.3	1.3	0.4
	Levofloxacin	2	>16	≤0.015–>16	44.0	10.8	45.3
DTR (125)	Imipenem-funobactam	8	>64	1–>64	16.0	20.8	63.2
	Imipenem	16	>128	4–>128	0	5.6	94.4
	Imipenem-relebactam	4	>64	1–>64	43.2	22.4	34.4
	Meropenem	16	>64	4–>64	0	14.4	85.6
	Meropenem-vaborbactam	16	>64	2–>64	NA	NA	NA
	Ceftazidime-avibactam	8	>128	1–>128	61.6	NA	38.4
	Cefiderocol	0.25	2	≤0.06–8	98.4	1.6	0
	Levofloxacin	>16	>16	2–>16	0	18.4	81.6

Abbreviations: % S, percent susceptible; % I, percent intermediate; % R, percent resistant; DTR, difficult-to-treat resistance; NA, not applicable ^a^ Imipenem-funobactam MICs were interpreted using 2026 CLSI M100 imipenem susceptible, intermediate, and resistant breakpoints as provisional breakpoints [11]. ^b^ NME species exclude *Proteus* species, *Providencia* species, and *Morganella morganii* which demonstrate intrinsic resistance to imipenem by a β-lactamase-independent mechanism.

## Data Availability

Data presented in this manuscript is available from the corresponding author upon reasonable request.

## Supplementary Materials

The following supporting information can be downloaded at: https://www.mdpi.com/article/10.3390/antibiotics15080733/s1, Table S1. *In vitro* activities of imipenem-funobactam and comparator agents against global clinical isolates of Enterobacterales collected in 2021 and 2022 categorized by infection source ^a^; Table S2. *In vitro* activities of imipenem-funobactam and comparator agents against global clinical isolates of non-*Morganellaceae* Enterobacterales (NME)^a^ collected in 2021 and 2022 categorized by infection source ^b^; Table S3. *In vitro* activities of imipenem-funobactam and comparator agents against global clinical isolates of *Acinetobacter baumannii* collected in 2021 and 2022 categorized by infection source; Table S4. *In vitro* activities of imipenem-funobactam and comparator agents against global clinical isolates of *Pseudomonas aeruginosa* collected in 2021 and 2022 categorized by infection source.
