# Supplementary material for: In Vitro Activity of Imipenem-Funobactam Against Clinical Isolates of Gram-Negative Bacilli
Source: Antibiotics (Basel). 2026 Jul 29;15(8):733. doi: 10.3390/antibiotics15080733 (PMC13510085; doi:10.3390/antibiotics15080733)
Supplement: Supplementary file 1 [file antibiotics-15-00733-s001.zip › antibiotics-4437434-supplementary.pdf]

Supplementary Data

Table S1. *In vitro* activities of imipenem-funobactam and comparator agents against global clinical isolates of Enterobacterales collected in 2021 and 2022 categorized by infection source[a].

		MIC, mg/L				MIC interpretation		
**Infection source (n)**	**Antimicrobial agent**	**MIC_{50}**	**MIC_{90}**	**MIC range**		**% S**	**% I**	**% R**
Blood (576)	Imipenem-funobactam[b]	0.12	1	≤0.03 – >64		92.0	4.2	3.8
	Imipenem	0.25	2	≤0.06 – >128		87.2	3.8	9.0
	Imipenem-relebactam	0.12	1	≤0.03 – >64		91.0	4.3	4.7
	Meropenem	≤0.06	0.12	≤0.06 – >64		93.9	0.3	5.7
	Meropenem-vaborbactam	0.03	0.12	≤0.008 – >64		96.4	0.2	3.5
	Ceftazidime-avibactam	0.12	0.5	≤0.06 – >128		97.6	NA	2.4
	Cefiderocol	0.12	1	≤0.06 – 8		99.8	0.2	0.0
	Levofloxacin	0.06	>16	≤0.015 – >16		69.3	4.9	25.9
Intra-abdominal (277)	Imipenem-funobactam	0.12	1	0.06 – 32		92.1	5.4	2.5
	Imipenem	0.25	2	≤0.06 – 128		88.4	4.7	6.9
	Imipenem-relebactam	0.12	1	0.06 – 32		92.1	4.3	3.6
	Meropenem	≤0.06	0.12	≤0.06 – >64		95.3	0.4	4.3
	Meropenem-vaborbactam	0.03	0.06	≤0.008 – >64		98.2	0.7	1.1
	Ceftazidime-avibactam	0.12	0.5	≤0.06 – >128		97.5	NA	2.5
	Cefiderocol	0.12	1	≤0.06 – 8		99.3	0.7	0.0
	Levofloxacin	0.06	16	≤0.015 – >16		72.6	5.4	22.0
Respiratory tract (531)	Imipenem-funobactam	0.25	1	0.06 – >64		91.7	4.3	4.0
	Imipenem	0.25	2	≤0.06 – >128		86.8	5.3	7.9
	Imipenem-relebactam	0.12	1	≤0.03 – >64		91.7	3.6	4.7
	Meropenem	≤0.06	0.12	≤0.06 – >64		93.2	0.8	6.0
	Meropenem-vaborbactam	0.03	0.06	≤0.008 – >64		96.6	0.6	2.8
	Ceftazidime-avibactam	0.25	1	≤0.06 – >128		96.0	NA	4.0
	Cefiderocol	0.12	1	≤0.06 – >32		99.4	0.2	0.4
	Levofloxacin	0.06	16	≤0.015 – >16		75.0	6.6	18.5
Skin and soft tissue (138)	Imipenem-funobactam	0.12	2	≤0.03 – 64		84.8	10.9	4.3
	Imipenem	0.25	2	≤0.06 – 128		81.9	8.7	9.4
	Imipenem-relebactam	0.12	2	≤0.03 – 64		84.8	9.4	5.8
	Meropenem	≤0.06	0.12	≤0.06 – >64		97.1	0.0	2.9
	Meropenem-vaborbactam	0.06	0.06	0.015 – 64		97.8	0.7	1.4
	Ceftazidime-avibactam	0.12	0.5	≤0.06 – >128		98.6	NA	1.4
	Cefiderocol	0.12	0.5	≤0.06 – 4		100	0.0	0.0
	Levofloxacin	0.06	16	≤0.015 – >16		74.6	5.8	19.6
Urinary tract (484)	Imipenem-funobactam	0.12	2	≤0.03 – >64		85.5	11.0	3.5
	Imipenem	0.25	2	≤0.06 – >128		81.2	11.8	7.0
	Imipenem-relebactam	0.12	2	≤0.03 – >64		85.5	10.7	3.7
	Meropenem	≤0.06	0.12	≤0.06 – >64		96.7	0.2	3.1
	Meropenem-vaborbactam	0.03	0.12	≤0.008 – >64		97.9	0.4	1.7
	Ceftazidime-avibactam	0.12	0.5	≤0.06 – >128		97.9	NA	2.1
	Cefiderocol	0.12	0.5	≤0.06 – 8		99.8	0.2	0.0
	Levofloxacin	0.12	16	≤0.015 – >16		68.4	6.6	25.0

Abbreviations: % S, percent susceptible; % I, percent intermediate; % R, percent resistant; NA, not applicable.

[a] Infection source was not available for two isolates.

[b] Imipenem-funobactam MICs were interpreted using 2026 CLSI M100 imipenem susceptible, intermediate, and resistant breakpoints as provisional breakpoints [11].

Table S2. *In vitro* activities of imipenem-funobactam and comparator agents against global clinical isolates of non-*Morganellaceae* Enterobacterales (NME)[a] collected in 2021 and 2022 categorized by infection source[b].

		MIC, mg/L				MIC interpretation		
**Infection source (n)**	**Antimicrobial agent**	**MIC_{50}**	**MIC_{90}**	**MIC range**		**% S**	**% I**	**% R**
Blood (531)	Imipenem-funobactam[c]	0.12	0.5	≤0.03 – >64		97.0	0.4	2.6
	Imipenem	0.12	1	≤0.06 – >128		91.9	1.5	6.6
	Imipenem-relebactam	0.12	0.5	≤0.03 – >64		95.7	1.1	3.2
	Meropenem	≤0.06	0.12	≤0.06 – >64		93.4	0.4	6.2
	Meropenem-vaborbactam	0.03	0.06	≤0.008 – >64		96.0	0.2	3.8
	Ceftazidime-avibactam	0.12	0.5	≤0.06 – >128		97.4	NA	2.6
	Cefiderocol	0.12	1	≤0.06 – 8		99.8	0.2	0.0
	Levofloxacin	0.06	>16	≤0.015 – >16		70.1	4.9	25.0
Intra-abdominal (255)	Imipenem-funobactam	0.12	0.25	0.06 – 32		97.6	0.8	1.6
	Imipenem	0.12	1	≤0.06 – 128		94.1	0.8	5.1
	Imipenem-relebactam	0.12	0.5	0.06 – 32		97.3	0.4	2.4
	Meropenem	≤0.06	0.12	≤0.06 – >64		94.9	0.4	4.7
	Meropenem-vaborbactam	0.03	0.06	≤0.008 – >64		98.0	0.8	1.2
	Ceftazidime-avibactam	0.12	1	≤0.06 – >128		97.3	NA	2.7
	Cefiderocol	0.12	1	≤0.06 – 8		99.2	0.8	0.0
	Levofloxacin	0.06	16	≤0.015 – >16		72.2	4.7	23.1
Respiratory tract (488)	Imipenem-funobactam	0.12	0.5	0.06 – >64		95.7	1.0	3.3
	Imipenem	0.25	1	≤0.06 – >128		91.6	1.8	6.6
	Imipenem-relebactam	0.12	0.5	≤0.03 – >64		95.1	1.0	3.9
	Meropenem	≤0.06	0.12	≤0.06 – >64		93.0	0.8	6.1
	Meropenem-vaborbactam	0.03	0.06	≤0.008 – >64		96.7	0.4	2.9
	Ceftazidime-avibactam	0.25	1	≤0.06 – >128		96.1	NA	3.9
	Cefiderocol	0.25	1	≤0.06 – >32		99.6	0.0	0.4
	Levofloxacin	0.06	16	≤0.015 – >16		75.8	6.4	17.8
Skin and soft tissue (106)	Imipenem-funobactam	0.12	0.5	≤0.03 – 8		99.1	0.0	0.9
	Imipenem	0.12	0.5	≤0.06 – 128		96.2	0.9	2.8
	Imipenem-relebactam	0.12	0.5	≤0.03 – 64		98.1	0.0	1.9
	Meropenem	≤0.06	≤0.06	≤0.06 – >64		97.2	0.0	2.8
	Meropenem-vaborbactam	0.03	0.06	0.015 – 64		98.1	0.9	0.9
	Ceftazidime-avibactam	0.12	0.5	≤0.06 – >128		99.1	NA	0.9
	Cefiderocol	0.25	0.5	≤0.06 – 4		100	0.0	0.0
	Levofloxacin	0.06	16	≤0.015 – >16		74.5	4.7	20.8
Urinary tract (371)	Imipenem-funobactam	0.12	0.25	≤0.03 – >64		97.8	0.3	1.9
	Imipenem	0.12	0.5	≤0.06 – >128		95.1	1.3	3.5
	Imipenem-relebactam	0.12	0.5	≤0.03 – >64		97.8	0.0	2.2
	Meropenem	≤0.06	≤0.06	≤0.06 – >64		96.2	0.3	3.5
	Meropenem-vaborbactam	0.03	0.06	≤0.008 – >64		97.8	0.3	1.9
	Ceftazidime-avibactam	0.12	0.5	≤0.06 – >128		97.8	NA	2.2
	Cefiderocol	0.12	1	≤0.06 – 8		99.7	0.3	0.0
	Levofloxacin	0.12	>16	≤0.015 – >16		66.0	7.5	26.4

Abbreviations: % S, percent susceptible; % I, percent intermediate; % R, percent resistant; NA, not applicable.

[a] NME species exclude *Proteus* species, *Providencia* species, and *Morganella morganii* which demonstrate intrinsic resistance to imipenem by a β-lactamase-independent mechanism [11].

[b] Infection source not available for one isolate.

[c] Imipenem-funobactam MICs were interpreted using 2026 CLSI M100 imipenem susceptible, intermediate, and resistant breakpoints as provisional breakpoints [11].

Table S3. *In vitro* activities of imipenem-funobactam and comparator agents against global clinical isolates of *Acinetobacter baumannii* collected in 2021 and 2022 categorized by infection source.

		**MIC, mg/L**				**MIC interpretation**		
**Infection source (n)**	**Antimicrobial agent**	**MIC_{50}**	**MIC_{90}**	**MIC range**		**% S**	**% I**	**% R**
Blood (200)	Imipenem-funobactam[a]	1	8	0.12 – >64		81.0	8.5	10.5
	Imipenem	64	128	0.12 – >128		35.0	0.0	65.0
	Imipenem-relebactam	32	>64	0.12 – >64		NA	NA	NA
	Meropenem	64	>64	≤0.06 – >64		34.5	0.0	65.5
	Meropenem-vaborbactam	32	>64	0.06 – >64		NA	NA	NA
	Ceftazidime-avibactam	32	>128	1 – >128		NA	NA	NA
	Cefiderocol	0.25	2	≤0.06 – >32		92.5	2.0	5.5
	Levofloxacin	8	>16	0.06 – >16		30.5	7.0	62.5
Intra-abdominal (44)	Imipenem-funobactam	1	4	0.06 – >64		86.4	4.5	9.1
	Imipenem	64	128	≤0.06 – >128		27.3	0.0	72.7
	Imipenem-relebactam	64	>64	0.06 – >64		NA	NA	NA
	Meropenem	64	>64	≤0.06 – >64		27.3	0.0	72.7
	Meropenem-vaborbactam	64	>64	≤0.008 – >64		NA	NA	NA
	Ceftazidime-avibactam	32	128	0.5 – >128		NA	NA	NA
	Cefiderocol	0.25	1	≤0.06 – 2		100	0.0	0.0
	Levofloxacin	8	>16	0.06 – >16		25.0	4.5	70.5
Respiratory tract (573)	Imipenem-funobactam	1	4	0.06 – >64		82.0	9.2	8.7
	Imipenem	64	128	0.12 – >128		24.4	0.5	75.0
	Imipenem-relebactam	64	>64	0.12 – >64		NA	NA	NA
	Meropenem	64	>64	≤0.06 – >64		24.3	0.2	75.6
	Meropenem-vaborbactam	64	>64	0.03 – >64		NA	NA	NA
	Ceftazidime-avibactam	32	>128	0.25 – >128		NA	NA	NA
	Cefiderocol	0.25	2	≤0.06 – >32		96.9	0.2	3.0
	Levofloxacin	8	>16	0.03 – >16		23.4	6.6	70.0
Skin and soft tissue (66)	Imipenem-funobactam	0.5	4	0.12 – >64		87.9	9.1	3.0
	Imipenem	32	128	0.12 – >128		39.4	0.0	60.6
	Imipenem-relebactam	32	>64	0.12 – >64		NA	NA	NA
	Meropenem	32	>64	0.12 – >64		39.4	0.0	60.6
	Meropenem-vaborbactam	32	>64	0.12 – >64		NA	NA	NA
	Ceftazidime-avibactam	32	128	2 – >128		NA	NA	NA
	Cefiderocol	0.5	2	≤0.06 – >32		97.0	0.0	3.0
	Levofloxacin	8	>16	0.03 – >16		39.4	6.1	54.5
Urinary tract (116)	Imipenem-funobactam	0.5	8	0.06 – >64		82.8	5.2	12.1
	Imipenem	32	128	0.12 – >128		45.7	0.0	54.3
	Imipenem-relebactam	32	>64	0.06 – >64		NA	NA	NA
	Meropenem	32	>64	0.12 – >64		44.0	1.7	54.3
	Meropenem-vaborbactam	32	>64	0.12 – >64		NA	NA	NA
	Ceftazidime-avibactam	32	>128	0.5 – >128		NA	NA	NA
	Cefiderocol	0.25	2	≤0.06 – >32		99.1	0.0	0.9
	Levofloxacin	4	>16	0.06 – >16		44.8	6.0	49.1

Abbreviations: % S, percent susceptible; % I, percent intermediate; % R, percent resistant; NA, not applicable.
[a] Imipenem-funobactam MICs were interpreted using 2026 CLSI M100 imipenem susceptible, intermediate, and resistant breakpoints as provisional breakpoints [11].

Table S4. *In vitro* activities of imipenem-funobactam and comparator agents against global clinical isolates of *Pseudomonas aeruginosa* collected in 2021 and 2022 categorized by infection source.

		MIC, mg/L				MIC interpretation		
**Infection source (n)**	**Antimicrobial agent**	**MIC_{50}**	**MIC_{90}**	**MIC range**		**% S**	**% I**	**% R**
Blood (166)	Imipenem-funobactam[a]	0.5	4	0.12 – >64		87.3	6.0	6.6
	Imipenem	2	16	0.25 – >128		77.1	5.4	17.5
	Imipenem-relebactam	0.5	2	0.12 – >64		92.2	4.2	3.6
	Meropenem	0.5	8	≤0.06 – >64		83.7	1.8	14.5
	Meropenem-vaborbactam	0.25	8	0.03 – >64		NA	NA	NA
	Ceftazidime-avibactam	2	8	0.5 – >128		95.8	NA	4.2
	Cefiderocol	0.25	1	≤0.06 – 8		99.4	0.6	0.0
	Levofloxacin	0.25	2	0.03 – >16		87.3	4.2	8.4
Intra-abdominal (74)	Imipenem-funobactam	0.5	2	0.25 – 16		90.5	5.4	4.1
	Imipenem	1	16	0.25 – 64		81.1	5.4	13.5
	Imipenem-relebactam	0.5	1	0.25 – 8		95.9	2.7	1.4
	Meropenem	0.5	2	≤0.06 – 64		90.5	1.4	8.1
	Meropenem-vaborbactam	0.25	2	0.06 – 64		NA	NA	NA
	Ceftazidime-avibactam	2	4	0.5 – 16		98.6	NA	1.4
	Cefiderocol	0.25	0.5	≤0.06 – 1		100	0.0	0.0
	Levofloxacin	0.25	4	≤0.015 – 16		83.8	5.4	10.8
Respiratory tract (536)	Imipenem-funobactam	0.5	8	0.12 – >64		82.8	6.0	11.2
	Imipenem	2	16	0.12 – >128		67.4	5.4	27.2
	Imipenem-relebactam	0.5	4	0.12 – >64		89.2	5.4	5.4
	Meropenem	0.5	16	≤0.06 – >64		73.3	8.4	18.3
	Meropenem-vaborbactam	0.5	16	≤0.008 – >64		NA	NA	NA
	Ceftazidime-avibactam	2	8	≤0.06 – >128		92.5	NA	7.5
	Cefiderocol	0.25	0.5	≤0.06 – >32		99.4	0.4	0.2
	Levofloxacin	0.5	16	≤0.015 – >16		75.4	6.9	17.7
Skin and soft tissue (90)	Imipenem-funobactam	0.5	2	0.25 – 64		92.2	3.3	4.4
	Imipenem	1	16	0.25 – 64		84.4	1.1	14.4
	Imipenem-relebactam	0.5	1	0.25 – 64		96.7	2.2	1.1
	Meropenem	0.25	4	≤0.06 – >64		88.9	4.4	6.7
	Meropenem-vaborbactam	0.25	2	0.06 – >64		NA	NA	NA
	Ceftazidime-avibactam	2	8	0.5 – >128		98.9	NA	1.1
	Cefiderocol	0.25	0.5	≤0.06 – 2		100	0.0	0.0
	Levofloxacin	0.25	2	0.06 – >16		85.6	7.8	6.7
Urinary tract (130)	Imipenem-funobactam	0.5	4	0.25 – >64		86.9	4.6	8.5
	Imipenem	2	16	0.5 – >128		81.5	3.1	15.4
	Imipenem-relebactam	0.5	2	0.25 – >64		91.5	0.8	7.7
	Meropenem	0.5	8	≤0.06 – >64		85.4	2.3	12.3
	Meropenem-vaborbactam	0.5	16	0.06 – >64		NA	NA	NA
	Ceftazidime-avibactam	2	8	1 – >128		91.5	NA	8.5
	Cefiderocol	0.25	0.5	≤0.06 – 4		100	0.0	0.0
	Levofloxacin	0.5	>16	0.06 – >16		73.8	4.6	21.5

Abbreviations: % S, percent susceptible; % I, percent intermediate; % R, percent resistant; NA, not applicable.
[a] Imipenem-funobactam MICs were interpreted using 2026 CLSI M100 imipenem susceptible, intermediate, and resistant breakpoints as provisional breakpoints [11].
